# Anxiety and Depression in Patients with Permanent Atrial Fibrillation: Prevalence and Associated Factors

**DOI:** 10.1155/2018/7408129

**Published:** 2018-02-19

**Authors:** Maria Polikandrioti, Ioannis Koutelekos, Georgios Vasilopoulos, Georgia Gerogianni, Maritsa Gourni, Sofia Zyga, George Panoutsopoulos

**Affiliations:** ^1^Department of Nursing, Faculty of Health and Caring Professions, Technological Educational Institute of Athens, Athens, Greece; ^2^Frederick University, Nicosia, Cyprus; ^3^Department of Nursing, Faculty of Human Movement and Quality of Life Sciences, University of Peloponnese, Sparta, Lakonia, Greece

## Abstract

Atrial fibrillation (AF) is an important public health problem that is increasing at an alarming rate, worldwide. The most common type is permanent AF followed by the paroxysmal and persistent AF. *Purpose*. This study was aimed at exploring anxiety and depression and the associated factors in patients with permanent AF. *Materials and Methods*. The sample of the study included 170 AF patients. Data collection was performed by the method of interview using the “Hospital Anxiety and Depression Scale” (HADS) to assess anxiety and depression and a questionnaire including patients' characteristics. *Results*. 70% of the participants were men, and 32.4% were above 70 years old. Furthermore, 34.9% of the patients had high levels of anxiety, and 20.2% had high levels of depression. Anxiety levels were statistically significantly associated with gender (*p*=0.022), age (*p*=0.022), educational level (*p*=0.025), years having the disease (*p*=0.005), and relations with nursing staff (*p*=0.040). Depression levels were statistically significantly associated with age (*p*=0.037), degree of information of the state of health (*p* < 0.001), years having the disease (*p* < 0.001), and relations with medical staff (*p*=0.041). *Conclusions*. Patients' characteristics are associated with anxiety and depression and need to be evaluated when treating this frequently encountered arrhythmia.

## 1. Introduction

Atrial fibrillation (AF) is the most common heart arrhythmia in clinical practice which is expected to increase dramatically within next decades due to the prolongation of life expectancy and the improvements in diagnosis [1]. According to estimates, AF currently affects over 6 million patients in Europe and approximately 2.3 million in the United States [2].

This arrhythmia is predominately a disease of the elderly [3] but remains asymptomatic in 30% to 44% of them [4]. AF is associated with increased morbidity and mortality [2, 3] rising risk of stroke and heart failure [4] and a significant burden for healthcare services with total annual treatment costs to be estimated at $6.65 billion in the US [5]. The most common type is permanent AF occurring in 40%–50% of patients, followed by the paroxysmal and persistent AF occurring in 20%–30% of cases [4]. Different types of AF are associated with different clinical patient profiles and long-term outcomes [4], thus requiring individualized approaches [6, 7].

The unpredictable nature of this arrhythmia with unexpected onset or recurrent events significantly affects all domains of a patient's life [8]. AF is associated with personal, clinical, socioeconomic implications [6, 7], diminished quality of life [7], and more frequent psychological disturbances compared to general population [8, 9].

Anxiety and depression are rising with the recurrent AF episodes [9], and are associated with symptom severity [10], with higher mortality rates [7], and with increased healthcare utilization [7, 10].

Regarding types of arrhythmia, depressive mood seems to be more common in persistent AF compared to paroxysmal AF [4]. The association between emotional burden and cardiac disease such as coronary artery disease, heart failure, or myocardial infarction is well cited in the literature. However, few studies have explored the psychological distress in forms of anxiety and depression in patients with permanent AF.

The *aim* of the present study was to explore the prevalence of anxiety and depression levels and the associated factors in patients with permanent AF.

## 2. Materials and Methods

### 2.1. Study Population

In the present study, 170 patients suffering from AF participated. Patients enrolled in the study were visiting the outpatient department of a public hospital for regular monitoring and follow-up.

This was a convenience sample. Patients were classified according to the current guidelines regarding type of AF [11]. Criteria for inclusion of patients in the study were (a) patients diagnosed with permanent AF and (b) ability to write and read the Greek language fluently. The exclusion criteria were (a) age <18 years, (b) inadequate follow-up, and (c) diagnosis of paroxysmal and persistent AF.

Written informed consent was obtained for all patients being interviewed.

Data collection was performed by the method of the interview using a questionnaire developed by the researchers so as to fully serve the purposes of the study. The data collected for each patient included sociodemographic characteristics (e.g., gender, age, education level, marital status, and number of children), clinical characteristics (e.g., years having the disease), and other self-reported characteristics (e.g., relations with the medical-nursing staff).

The study was approved by the Medical Research Ethics Committee of the hospital and conducted in accordance with the Declaration of Helsinki (1989) of the World Medical Association.

### 2.2. Hospital Anxiety and Depression (HAD) Scale

The “Hospital Anxiety and Depression Scale (HADS)” was used to evaluate depression and anxiety of patients. This scale was proposed in 1983 by Zigmond and Snaith [12]. The scale consists of 14 questions that assess how patients felt during the previous week. Patients are able to answer every question in a 4-point Likert scale from 0 to 3. Seven of 14 questions assess the level of depression, and the other seven evaluate the anxiety level. Scores attributed to questions are summed separately for anxiety and depression, leading to two scores with range 0–21. Higher score indicates higher levels of anxiety and depression, respectively. In addition, it is widely used in the literature with the following categorization: score 0–7 indicating no stress or depression, score 8–10 indicating moderate levels of anxiety or depression, and score >11 indicating high levels of anxiety or depression. In Greek population, the HADS has been translated and tested for its validity and reliability by Mystakidou et al. [13].

### 2.3. Statistical Analysis

Categorical variables are presented by absolute and relative frequencies (percentages), and continuous variables are presented by median and interquartile range. To test the existence of association between levels of anxiety/depression and patient characteristics, the Kruskal–Wallis test and the *χ*^2^ test of independence were used. Multinomial logistic regression was performed to estimate the effect of patient characteristics on the levels of anxiety/depression (dependent variable). The results are presented with odds ratio (OR) and 95% confidence intervals. The level of statistical significance was set to *a* = 5%. The analysis was performed with the statistical package SPSS, version 22 (SPSS Inc., Chicago, IL, USA).

## 3. Results

### 3.1. Patients' Characteristics

From Table 1, we observe that 70% of the patients were men and approximately 63% over 60 years. Moreover, 74.1% of the sample was married, 51.8% had primary education, 62.4% were pensioners, 67.1% lived in Attica, and 48.2% had two children. Furthermore, 43.5% of the patients had some other diseases, while the majority of the sample reported to be “very” informed about their problem (52.4%). Almost half of the sample had the disease less than 2 years (48.2%). Lastly, the majority reported to have “very good” relations with nursing and medical staff, 72.9% and 70%, respectively.

### 3.2. Levels of Anxiety/Depression

From Table 2, we conclude that 34.9% and 20.2% of participants had high levels of anxiety and depression, respectively.

### 3.3. Association between Patients' Characteristics and Anxiety Levels

Table 3 presents the association between anxiety levels and patients' characteristics. Anxiety levels were statistically significantly associated with gender (*p*=0.022), age (*p*=0.022), educational level (*p*=0.025), years having the disease (*p*=0.005), and relations with nursing staff (*p*=0.040). Specifically, the percentage of female patients with high levels of anxiety (49%) was higher than male patients with high levels of anxiety (28.8%). Elderly patients were more likely to have high levels of anxiety (42.6%) than patients 61–70 years old (30.8%) and patients below 60 years old (31.7%). Moreover, patients with university educational level or those having primary education were more likely to have high levels of anxiety (44% and 40.9%, resp.) than patients with secondary education level (30.8%). The percentage of pensioner patients with high levels of anxiety (41.9%) was higher than employee patients with high levels of anxiety (23.4%). Patients having the disease for 6–10 years were more likely to have high levels of anxiety (45.5%) than those having the disease for 2–5 years (32.6%) or less than 2 years (30.5%). Lastly, patients who reported to have “moderate/bad” relations with nursing staff or “good” relations were more likely to have high levels of anxiety (50% and 41.5%, resp.) than patients who reported to have “very good” relations (31.4%).

### 3.4. Association between Patients' Characteristics and Depression Levels

Table 4 presents the association between depression and patients' characteristics. Depression levels were statistically significantly associated with age (*p*=0.037), degree of information of the state of health (*p* < 0.001), years having the disease (*p* < 0.001), and relations with medical staff (*p*=0.041). Specifically, elderly patients were more likely to have high levels of depression (33.3%) than patients 61–70 years old (15.7%) and patients below 60 years old (12.7%). Moreover, patients who were “a little/not at all” informed for the state of their health were more likely to have high levels of depression (40%) than patients who were “enough” informed (18%) or “very” informed (17.2%). Patients having the disease 6–10 years were more likely to have high levels of depression (44.2%) than those having the disease for 2–5 years (9.3%) or less than 2 years (13.4%). Lastly, patients who reported to have “moderate/bad” relations with medical staff or “good” relations were more likely to have high levels of depression (30% and 31.7%, resp.) than patients who reported to have “very good” relations (15.4%).

### 3.5. Effect of Patients' Characteristics on the Levels of Anxiety/Depression

Multinomial logistic regression was performed in order to assess the effect of independent factors associated with anxiety and depression. From Table 5, we conclude that patients aged above 70 years old have 77% less chances to experience moderate levels of anxiety related to low levels, than those aged below 60 years old (OR = 0.23, 95% CI: 0.06–0.83, *p*=0.026). Pensioner patients have 3.21 times higher chances to experience high levels of anxiety related to low levels, than employee patients (OR = 3.21, 95% CI: 1.03–10.01, *p*=0.044). Patients having “good” relations with nursing staff have 3.25 and 3.89 times higher chances to experience moderate and high levels of anxiety, respectively, related to low levels, than patients having “very good” relations with nursing staff (OR = 3.25, 95% CI: 1.07–9.83, *p*=0.037 and OR = 3.89, 95% CI: 1.32–11.41, *p*=0.013). Moreover, patients having “bad” relations with nursing staff have 6.58 times higher chances to experience high levels of anxiety related to low levels, than patients having “very good” relations with nursing staff (OR = 6.58, 95% CI: 1.09–39.68, *p*=0.040).

Regarding the impact of factors on the levels of depression (Table 6), we conclude that patients who were “enough” informed of the state of their health have 7.24 times higher chances to experience moderate levels of depression, and those who were “less/not at all” informed have 3.77 times higher chances to experience high levels of depression related to low levels, than those who were very informed (OR = 7.24, 95% CI: 2.45–21.44, *p*=0.001 and OR = 3.77, 95% CI: 1.04–13.71, *p*=0.044, resp.). Patients having the disease for 6–10 years have 6.31 times higher chances to experience high levels of depression related to low levels than patients having the disease less than 2 years (OR = 6.31, 95% CI: 2.10–19.00, *p*=0.001). Moreover, patients having “good” relations with medical staff have 5.18 times higher chances to experience high levels of depression related to low levels than patients having “very good” relations (OR = 5.18, 95% CI: 1.70–15.77, *p*=0.004).

Convenience sampling was one of the limitations of this study. Other limitations were related to the study design which was cross-sectional and not longitudinal. Anxiety and depression symptoms were measured only once, and we do not know whether they changed over time. Additionally, no structured psychiatric interviews were conducted, and no data were collected about receiving medicine (anxiolytics and antidepressants) or any self-treatments for anxiety or depression.

Despite limitations, these data have important clinical implications for management of permanent AF patients.

## 4. Discussion

According to the results of the present study, 34.9% and 20.2% of the patients had high levels of anxiety and depression, respectively. Thrall et al. [14], in baseline assessment of AF patients, showed symptoms of depression and state-trait anxiety in 38%, 28%, and 38%, respectively. After 6 months, anxiety and depression remained high in one-third of AF patients. Thompson et al. [10] demonstrated 39.4% and 16.9% mild to moderate and severe depression, respectively, in 378 AF patients.

Meanwhile, von Eisenhart Rothe et al. in 2015 showed that depressed mood was associated with AF symptom burden over 6 months after adjustment for perceived frequency and duration of AF episodes, chronic obstructive pulmonary disorder, and sex [15]. One year earlier, the same researcher indicated that persistent AF patients suffered more severe depressed mood when compared to paroxysmal AF patients with similar symptom burden [9]. Strikingly more, von Eisenhart Rothe et al. [16], who explored in 2013, 334 paroxysmal AF patients without significant concomitant heart diseases, illustrated that physicians rated patients' health-related quality higher than patients did. Therefore, evaluating the gap between physicians' estimations and AF patients' self-ratings regarding quality of life is another significant area related to this disease.

Anxiety is the main response to diagnosis of AF in patients without other associated comorbidities [17]. More in detail, Lane et al. [17] showed prevalence of elevated state anxiety in 38.5%, 30.9%, and 35.7% AF patients at baseline (diagnosis) and at 6 and 12 months, respectively. Moreover, AF patients when compared to the hypertensive ones experience higher trait anxiety (38% versus 22%) [14]. However, treating AF may improve symptom severity but does not reduce anxiety and depression [10].

Given that anxiety and depression frequently co-occur in AF individuals, it is essential to optimize the management of this comorbidity [18].

The result that female patients experienced high levels of anxiety is in line with Trovato et al. [19], who demonstrated greater psychological stress in women with stable AF. The present study showed no significant association between women and depression which contradicts results by Dabrowski et al. [20], indicating higher levels of depression in women, along with sleep problems and physical limitations, irrespective of AF type. The female preponderance to anxiety is partially explained by the well-documented sex differences in the pattern and outcomes of cardiac disease ranging from genetic factors to differences in daily living or health behaviors, delays in responding to symptoms, and several others [21]. Accordingly, female may be more vulnerable to distress mainly those living alone as a result of being divorced/widowed or having comorbid conditions. Taking into consideration that women may have worse outcomes [22] or increased risk of death when compared to male counterparts (90% versus 50%), it becomes apparent that women should undergo diagnostic evaluation and anxiety alleviation [23].

Data also revealed that elderly AF patients were more likely to have high levels of depression and anxiety. Several explanations may account for this observed increase such as physical impairment, unhealthy lifestyle, poor treatment adherence [24], or cognitive impairment [25, 26]. Awareness that AF prevalence is increasing markedly with advancing age as almost 70% of patients are between 65 and 85 years of age undoubtedly allows more targeted treatment approach in this rapidly growing population [24–26].

Patients having the disease 6–10 years were more likely to have high levels of anxiety and depression. It is widely known that chronic illness keeps up with psychological disorders, thus increasing morbidity and mortality [27]. Therefore, duration of the cardiac disease is associated with depression. For instance, in heart failure patients, the longer the disease duration, the higher the probability of being depressed [28]. Unfortunately, depression in 50%–70% of patients with chronic illness remains undiagnosed due to several reasons such as lack of symptom awareness, confused clinical picture, and reluctance to admit psychological burden [27].

Moreover, high levels of depression had patients who were a little or not at all informed about their health and those who had moderate or bad relations with medical staff. It goes without saying that mutual collaboration with health professionals is enhancing patients' understanding about the therapeutic regimen. Effective communication enables health professionals to understand patients' beliefs, attitudes, and emotional challenges [29]. AF patients, who obtain accurate and elaborate information, usually report fewer symptoms, perceive greater control over the disease, and experience less emotional distress [30, 31]. Optimal treatment outcomes demand addressing information needs of patients or perceptions about the disease (consequences and controllability of the disease) [32]. It should be stressed that the relation between depression, anxiety, and AF is complex. More in detail, AF may trigger depression and anxiety, whereas depression and anxiety may create such an environment where AF will be more easily established [33]. Therefore, care of AF patient demands a structured plan for follow-up [11].

## 5. Conclusions

Both anxiety and depression were associated with age, years having the disease, and relations with nursing and medical staff. Separately, anxiety levels were higher in female, those with university or primary education and in employees, whereas depression levels were higher in elderly patients or those below 60 years old and in patients who reported to be “a little/not at all” informed about their health.

Based on the findings presented, it is suggested that assessing anxiety and depression is fundamental to the development of appropriate interventions that address this psychological burden of patients living with AF. Additionally, more awareness of factors associated with anxiety and depression in AF is supposed to help clinical decision-making.

## Figures and Tables

**Table 1:** Patients' characteristics (*N* = 170).

	*n* (%)
Gender (male)	119 (70.0%)
Age (years)	
<50	26 (15.3%)
51–60	37 (21.8%)
61–70	52 (30.6%)
>70	55 (32.4%)
Marital status	
Married	126 (74.1%)
Single	44 (25.9%)
Educational level	
Primary	88 (51.8%)
Secondary	56 (32.9%)
University	26 (15.3%)
Job	
Employee	64 (37.6%)
Pensioner/household	106 (62.4%)
Place of residence	
Attica	114 (67.1%)
Other places	56 (32.9%)
Number of children	
None	19 (11.2%)
One	35 (20.6%)
Two	82 (48.2%)
More than two	34 (20.0%)
Other diseases (yes)	74 (43.5%)
Informed of the state of their health	
Very	89 (52.4%)
Enough	61 (35.9%)
Less/not at all	20 (11.8%)
Years having the disease	
<2	82 (48.2%)
2–5	44 (25.9%)
6–10	44 (25.9%)
Relations with nursing staff	
Very good	124 (72.9%)
Good	34 (20.0%)
Moderate/bad	12 (7.1%)
Relations with medical staff	
Very good	119 (70.0%)
Good	41 (24.1%)
Moderate/bad	10 (5.9%)

**Table 2:** Levels of anxiety/depression of patients (*N* = 170).

	*Ν* (%)
*Anxiety*	
Low levels of anxiety (score range: 0–7)	65 (38.5%)
Moderate levels of anxiety (score range: 8–10)	45 (26.6%)
High levels of anxiety (score range: 11–21)	59 (34.9%)
*Depression*	
Low levels of depression (score range: 0–7)	108 (64.3%)
Moderate levels of depression (score range: 8–10)	26 (15.5%)
High levels of depression (score range: 11–21)	34 (20.2%)

**Table 3:** Association between patients' characteristics and anxiety levels.

Characteristics	Low levels, *N* (%)	Moderate levels, *N* (%)	High levels, *N* (%)	*p* value
Gender				
Male	47 (39.8%)	37 (31.4%)	34 (28.8%)	**0.022**
Female	18 (35.3%)	8 (15.7%)	25 (49.0%)	
Age				
≤60	19 (30.2%)	24 (38.1%)	20 (31.7%)	**0.022**
61–70	21 (40.4%)	15 (28.8%)	16 (30.8%)	
>70	25 (46.3%)	6 (11.1%)	23 (42.6%)	
Status				
Married	47 (37.6%)	33 (26.4%)	45 (36.0%)	0.876
Single	18 (40.9%)	12 (27.3%)	14 (31.8%)	
Educational level				
Primary	36 (40.9%)	16 (18.2%)	36 (40.9%)	**0.025**
Secondary	23 (41.1%)	21 (37.5%)	12 (21.4%)	
University	6 (24.0%)	8 (32.0%)	11 (44.0%)	
Job				
Employee	23 (35.9%)	26 (40.6%)	15 (23.4%)	**0.003**
Pensioner/household	42 (40.0%)	19 (18.1%)	44 (41.9%)	
Place of residence				
Attica	43 (37.7%)	33 (28.9%)	38 (33.3%)	0.604
Other places	22 (40.0%)	12 (21.8%)	21 (38.2%)	
Number of children				
None	7 (36.8%)	8 (42.1%)	4 (21.1%)	0.368
One	13 (37.1%)	11 (31.4%)	11 (31.4%)	
More than one	45 (39.1%)	26 (22.6%)	44 (38.3%)	
Other diseases				
Yes	26 (35.6%)	16 (21.9%)	31 (42.5%)	0.179
No	39 (40.6%)	29 (30.2%)	28 (29.2%)	
Informed of the state of their health				
Very	39 (44.3%)	23 (26.1%)	26 (29.5%)	0.391
Enough	18 (29.5%)	18 (29.5%)	25 (41.0%)	
Less/not at all	8 (40.0%)	4 (20.0%)	8 (40.0%)	
Years having the disease				
<2	32 (39.0%)	25 (30.5%)	25 (30.5%)	**0.005**
2–5	18 (41.9%)	11 (25.6%)	14 (32.6%)	
6–10	15 (34.1%)	9 (20.5%)	20 (45.5%)	
Relations with nursing staff				
Very good	56 (45.2%)	30 (24.2%)	38 (30.6%)	**0.040**
Good	7 (21.2%)	12 (36.4%)	14 (42.4%)	
Moderate/bad	2 (16.7%)	3 (25.0%)	7 (58.3%)	
Relations with medical staff				
Very good	53 (44.9%)	28 (23.7%)	37 (31.4%)	0.128
Good	10 (24.4%)	14 (34.1%)	17 (41.5%)	
Moderate/bad	2 (20.0%)	3 (30.0%)	5 (50.0%)	

Low-level score: 0–7; moderate-level score: 8–10; high-level score: 11–21.

**Table 4:** Association between patients' characteristics and depression levels.

Characteristics	Low levels, *N* (%)	Moderate levels, *N* (%)	High levels, *N* (%)	*p* value
Gender				
Male	77 (65.8%)	17 (14.5%)	23 (19.7%)	0.808
Female	31 (60.8%)	9 (17.6%)	11 (21.6%)	
Age				
≤60	47 (74.6%)	8 (12.7%)	8 (12.7%)	**0.037**
61–70	34 (66.7%)	9 (17.6%)	8 (15.7%)	
>70	27 (50.0%)	9 (16.7%)	18 (33.3%)	
Status				
Married	80 (64.5%)	18 (14.5%)	26 (21.0%)	0.814
Single	28 (63.6%)	8 (18.2%)	8 (18.2%)	
Educational level				
Primary	54 (61.4%)	12 (13.6%)	22 (25.0%)	0.534
Secondary	39 (69.6%)	9 (16.1%)	8 (14.3%)	
University	15 (62.5%)	5 (20.8%)	4 (16.7%)	
Job				
Employee	45 (70.3%)	7 (10.9%)	12 (18.8%)	0.355
Pensioner/household	63 (60.6%)	19 (18.3%)	22 (21.2%)	
Place of residence				
Attica	72 (63.7%)	21 (18.6%)	20 (17.7%)	0.195
Other places	36 (65.5%)	5 (9.1%)	14 (25.5%)	
Number of children				
None	14 (73.7%)	3 (15.8%)	2 (10.5%)	0.567
One	24 (68.6%)	3 (8.6%)	8 (22.9%)	
More than one	70 (61.4%)	20 (17.5%)	24 (21.1%)	
Other diseases				
Yes	46 (63.9%)	9 (12.5%)	17 (23.6%)	0.489
No	62 (64.6%)	17 (17.7%)	17 (17.7%)	
Informed of the state of their health				
Very	66 (75.9%)	6 (6.9%)	15 (17.2%)	**<0.001**
Enough	32 (52.5%)	18 (29.5%)	11 (18.0%)	
Less/not at all	10 (50.0%)	2 (10.0%)	8 (40.0%)	
Years having the disease				
<2	56 (68.3%)	15 (18.3%)	11 (13.4%)	**<0.001**
2–5	31 (72.1%)	8 (18.6%)	4 (9.3%)	
6–10	21 (48.8%)	3 (7.0%)	19 (44.2%)	
Relations with nursing staff				
Very good	84 (68.3%)	18 (14.6%)	21 (17.1%)	0.309
Good	17 (51.5%)	7 (21.2%)	9 (27.3%)	
Moderate/bad	7 (58.3%)	1 (8.3%)	4 (33.3%)	
Relations with medical staff				
Very good	82 (70.1%)	17 (14.5%)	18 (15.4%)	**0.041**
Good	19 (46.3%)	9 (22.0%)	13 (31.7%)	
Moderate/bad	7 (70.0%)	0 (0.0%)	3 (30.0%)	

Low-level score: 0–7; moderate-level score: 8–10; high-level score: 11–21.

**Table 5:** Effect of patients' characteristics on the levels of anxiety.

Characteristics	Anxiety levels (reference category: low levels)			
	Moderate levels		High levels	
	OR (95% CI)	*p* value	OR (95% CI)	*p* value
Gender				
Male	Ref. cat.	—	Ref. cat.	—
Female	0.72 (0.26–2.03)	0.536	2.16 (0.91–5.14)	0.081
Age				
≤60	Ref. cat.	—	Ref. cat.	—
61–70	0.7 (0.23–2.12)	0.527	0.51 (0.16–1.56)	0.237
>70	0.23 (0.06–0.83)	**0.026**	0.46 (0.15–1.39)	0.168
Educational level				
Primary	Ref. cat.	—	Ref. cat.	—
Secondary	1.5 (0.61–3.71)	0.380	0.55 (0.22–1.38)	0.202
University	1.85 (0.47–7.25)	0.378	3.37 (0.91–12.53)	0.069
Job				
Employee	Ref. cat.	—	Ref. cat.	—
Pensioner/household	0.81 (0.28–2.39)	0.706	3.21 (1.03–10.01)	**0.044**
Years having the disease				
<2	Ref. cat.	—	Ref. cat.	—
2–5	0.69 (0.26–1.87)	0.467	0.89 (0.33–2.37)	0.812
6–10	1.23 (0.4–3.78)	0.714	1.88 (0.71–5.01)	0.205
Relations with nursing staff				
Very good	Ref. cat.	—	Ref. cat.	—
Good	3.25 (1.07–9.83)	**0.037**	3.89 (1.32–11.41)	**0.013**
Moderate/bad	1.92 (0.27–13.49)	0.512	6.58 (1.09–39.68)	**0.040**
Other diseases				
Yes	Ref. cat.	—	Ref. cat.	—
No	0.88 (0.36–2.17)	0.780	0.95 (0.42–2.16)	0.912

Low-level score: 0–7; moderate-level score: 8–10; high-level score: 11–21.

**Table 6:** Effect of patients' characteristics on the levels of depression.

Characteristics	Depression levels (reference category: low levels)			
	Moderate levels		High levels	
	OR (95% CI)	*p* value	OR (95% CI)	*p* value
Age				
≤60	Ref. cat.	—	Ref. cat.	—
61–70	1.92 (0.60–6.15)	0.273	1.07 (0.32–3.58)	0.911
>70	3.22 (0.94–11.09)	0.064	2.71 (0.87–8.39)	0.084
Informed of the state of their health				
Very	Ref. cat.	—	Ref. cat.	—
Enough	7.24 (2.45–21.44)	**0.001**	0.98 (0.32–3.04)	0.973
Less/not at all	1.68 (0.28–10.08)	0.573	3.77 (1.04–13.71)	**0.044**
Years having the disease				
<1	Ref. cat.	—	Ref. cat.	—
2–5	1.35 (0.45–4.03)	0.588	0.53 (0.13–2.12)	0.368
6–10	0.66 (0.15–2.98)	0.590	6.31 (2.10–19.00)	**0.001**
Relations with medical staff				
Very good	Ref. cat.	—	Ref. cat.	—
Good	1.52 (0.53–4.39)	0.441	5.18 (1.70–15.77)	**0.004**
Moderate/bad	—^∗^	—	1.84 (0.31–10.87)	0.502
Other diseases				
Yes	Ref. cat.	—	Ref. cat.	—
No	1.84 (0.66–5.13)	0.241	1.06 (0.42–2.67)	0.903

^∗^Data are not presented because the sample size was too small to assess the effect; low-level score: 0–7; moderate-level score: 8–10; high-level score: 11–21.
